# A link to the past: classical phage ISP infects the recently described *Staphylococcus borealis* species

**DOI:** 10.1186/s12985-025-02935-z

**Published:** 2025-09-29

**Authors:** Arthur Kruse Sørensen, Jeroen Wagemans, Md Sabuj Hosen, Hermoine Jean Venter, Jorunn Pauline Cavanagh, Klara Stensvåg, Rob Lavigne, Gabriel Magno de Freitas Almeida

**Affiliations:** 1 The Norwegian College of Fishery Science, Faculty of Biosciences, Fisheries and Economics, UiT - The Arctic University of Norway, Tromsø, Norway; 2 Department of Biosystems, KU Leuven, Leuven, Belgium; 3 Research Group for Child and Adolescent Health, Department of Clinical Medicine, UiT- The Arctic University of Norway, Tromsø, Norway; 4 Centre for New Antibacterial Strategies, UiT The Arctic University of Norway, Tromsø, Norway

**Keywords:** *Staphylococcus borealis*, Phages, Host range, Phage ISP, Phage therapy, *Staphylococci*

## Abstract

Staphylococcal infections, caused by a large variety of species within the *Staphylococcus* genus, are a threat to human health. Although antibiotics are the current choice of treatment for these infections, bacteriophage (phage) therapy has been used with success against *Staphylococcus* since the dawn of phage therapy. In 2020 a new coagulase-negative species named *Staphylococcus borealis* was described in Norway. In this study, we focused on understanding phage infections in *S. borealis*. Methods: First, we predict the presence of prophages and phage-defence mechanisms in the genomes of a collection of twelve *S. borealis* strains by bioinformatics. We also attempted to isolate *S. borealis*-infecting phages from Norwegian samples and tested the host-range of three known staphylococcal phages against a panel of fifty Norwegian staphylococcal strains. Results: The presence of prophages and phage defence systems was verified in all tested *S. borealis* strains. No local Norwegian phages could be obtained in a phage isolation attempt targeted towards *S. borealis*. The host range analysis shows that phage ISP, originally isolated in the 1920s and still used for phage therapy to date, can infect *S. capitis* and the *S. borealis* Hus23 strain. Phage ISP was shown to limit *S. borealis* Hus23 growth in liquid cultures and lower the formation of biofilm by the bacterium. The efficiency of plating of phage ISP can be improved by repeated passages in the new *S. borealis* Hus23 host. Conclusions: here we expand the known host range of the traditional phage ISP by showing that it also infects *S. borealis* and can be adapted to the new host by serial passages, showcasing the flexibility of phages as an antimicrobial strategy.

**Supplementary Information:**

The online version contains supplementary material available at 10.1186/s12985-025-02935-z.

## Background

*Staphylococcus* is a genus of Gram-positive bacteria commensal to animal skin, mucous membranes and nasal cavities. Bacteria from this genus are opportunistic and are known to cause clinical indications like ulcers, soft tissue infections and sepsis [1]. *S. aureus*, a well-known species of the genus, is a coagulase-positive bacterium listed as one of the World Health Organization ESKAPE pathogens. Species from the coagulase-negative group, such as *S. epidermidis* and *S. haemolyticus*, are also clinically relevant, often related to infections following colonisation of medical devices [2]. Spread of antimicrobial resistance, zoonotic potential and emergent infectious disease dynamics, in addition to progress of modern medicine and an ageing population, are factors that contribute to an increase in staphylococcal threat to humankind [3]– [4].

A new coagulase-negative *Staphylococcus* species was described in North Norway in 2020. During a study focused on a large cohort of *S. haemolyticus* isolates from humans, Pain et al. discovered a subset of strains that had peculiar traits. In-depth genomic and phenotypic analyses led to the conclusion that these isolates were in fact a novel species, named *S. borealis* [5]. *S. borealis’* 16 S rRNA sequences and MALDI-TOF profiles have a high similarity with those from *S. haemolyticus*, which might have been leading to misidentification of isolates in the past. In fact, after the description of the new species, phylogeny identified three additional deposited draft genome sequences that also belonged to the *S. borealis* group [5]. Twelve strains isolated from bovines in Poland and Canada which had originally been classified as *S. haemolyticus* due to their high 16 S rRNA identity were later reanalysed based on whole genome sequencing and shown to possess 97% nucleotide identity to *S. borealis* (opposed to 88% identity to *S. haemolyticus*) [6]. In the same study, the authors found genomes deposited in GenBank showing that two additional *S. borealis* strains were also isolated from pigs in China and the Netherlands. All this evidence reveals that *S. borealis* is not limited to the North, can be found in human and animal samples, and is likely widely distributed. The high similarity between the 16 S rRNA genes and the MALDI-TOF profiles of *S. borealis* and *S. haemolyticus* indicates that part of the *S. haemolyticus* strains worldwide may indeed be *S. borealis*. An analysis of 129 *S. borealis* isolates has shown that this pathogen is more prevalent in middle-aged to elderly males, is able to form biofilms in vitro, and has lower resistance toward methicillin and penicillin when compared to clinical *S. haemolyticus* isolates [7]. Antibiotic therapy is the current treatment of choice for staphylococcal infections. In the last decades, the number of infection cases has increased, followed by the spread of antimicrobial resistance in clinical strains [3]– [4]. This makes finding alternative or complementary strategies to antibiotic usage urgent. Bacteriophages (phages) against staphylococci are known and possess different host ranges. Phage therapy has been used against staphylococci since its golden age. In fact, the first reported use of phage therapy in humans was against staphylococcal skin disease [8]– [9]. Treatment of staphylococcal diseases with phages before the antibiotic era was likely common. Dr Cruz, one pioneer of phage therapy in South America during the early 20th century, considered phages the “*first order therapeutic agent for staphylococcal septicaemia*” in the 1930 s [10]. Since then, staphylococcal species have been extensively targeted in vitro, in vivo and by clinical phage therapy approaches [11]– [12]. Recently, one-third of the first 100 consecutive cases of personalized phage therapy in Belgium involved *Staphylococcus* infections [13]. Staphylococcal phages infect different species within the genus, including *S. haemolyticus* and other coagulase-negative staphylococci [14]. To date, no specific phages or recorded phage infections in *S. borealis* strains have been described [15].

In this study, we first aimed at isolating phages capable of infecting *S. borealis* from Norwegian sources. After failing in this attempt, we tested the host range of three known *Staphylococcus* phages against a collection of staphylococcal isolates from Norway, showing for the first time that one of the tested phages (ISP) can infect a *S. borealis* strain (Hus23). The efficacy of phage ISP against *S. borealis* Hus23 was improved in vitro by repeated passages of the phage in the *S. borealis* host, showing that adapting the phage is a realistic process. In conclusion, although finding phages specific for *S. borealis* may be difficult, known staphylococci phages could become suitable candidates for phage therapy against this species in case of need.

## Methods

### Bacteria and phage strains used

A list of the bacteria strains used can be seen in Table 1. Bacterial growth was made in TSB media (Difco) under agitation at 37 °C. Phage ISP [16]phage Huma [17] and phage Romulus [18] were kindly donated by KU Leuven (Belgium). All three phages were propagated using *S. aureus* PS47. All phage experiments were made in TSB media supplemented with 1 mM of CaCl_2_ (Merck, Darmstadt, Germany) and 1 mM of MgSO_4_ (Merck, Darmstadt, Germany). Phage stocks and phage titrations were made using double-agar plates. Double-agar plates were made by mixing 200 µl of turbid overnight cultures of the desired host with three millilitres of soft-TSB (0.7% w/v agar) media, followed by briefly vortexing and pouring the whole mixture on top of a TSB agar plate.

**Table 1:** Host range results

Species	ID/code	Phage ISP	Phage Huma	Phage Romulus
*S. aureus*	63-36	7.00E+05	+	+
	63-37	+	+	+
	63-38	1.70E+06	+	+
	63-39	-	-	-
	69-01	+	+	+
	ATCC 6538	-	-	-
	ATCC PS47	3.60E+06	4.00E+05	+
	ATCC 9144	-	-	-
	51-03	-	-	-
*S.borealis*	51-48	-	-	-
	AHUS3	-	-	-
	HNT1	-	-	-
	HNT4	-	-	-
	HNT11	-	-	-
	HUS3	-	-	-
	HUS23	2.00E+05	-	-
	SSHF2	-	-	-
	SSHF10	-	-	-
	SSHF14	-	-	-
	SSHF15	-	-	-
	UNN28	-	-	-
*S. capitis*	59-1	2.00E+06	-	-
*S.epidermidis*	59-2	-	-	-
	59-4	-	-	-
	RP62A	-	-	-
	5179R1	-	-	-
*S.haemolyticus*	51-16	-	-	-
	51-13	-	-	-
	51-07	-	-	-
	51-27	-	-	-
	51-32	-	-	-
	51-34	-	-	-
	51-42	-	-	-
	54-58	-	-	-
	54-46	-	-	-
	51-21	-	-	-
	51-41	-	-	-
	57-47	-	-	-
	53-37	-	-	-
	51-46	-	-	-
	57-26	-	-	-
	53-36	-	-	-
	53-50	-	-	-
	57-22	-	-	-
	25-63	-	-	-
	53-62	-	-	-
	53-20	-	-	-
	57-08	-	-	-
	57-25	-	-	-
	53-38 (wild type)	-	-	-
	53-38.7Δ*CapA*	-	-	-
	53-38.16Δ*CapA*	-	-	-
	53-38.26Δ*CapA*	-	-	-
	53-38.5Δ*CapA-O*	-	-	-
	53-38.14Δ*Cap-O*	-	-	-
*S.lungdunensis*	59-3	-	-	-

Negative results are indicated with a minus sign. Positive results are marked in green. A plus sign marks the positive samples in which only Lysis (no countable plaques) appeared. In the occasions in which countable plaques were seen, the titre of the phage is shown in pfu/ml. Strains ATCC 6538, 9144 and PS47 are from the ATCC. All other strains are from the UiT collection. Note that *S. haemolyticus* strain 53 − 38 was tested as wild type and as five capsule-related mutants

### Bioinformatics analyses

Prophages in the *S. borealis* strains were identified using version 3.0 of PHASTEST online tool [19]. Anti-phage defence mechanisms were investigated using PADLOC [20].

### Phage isolation strategies

Isolation attempts were made by direct plating and by preparation of enrichment cultures. For direct plating we mixed 200 µl of a given filtered sample with 200 µl of the host to be used, added the mixture to three millilitres of soft-TSB and poured all on top of TSB plates. Enrichment cultures were made by mixing 0.5 ml of a turbid culture of the host with 3.5 ml of a sample and 1 ml of 5x TSB media. Enrichment cultures were incubated overnight and sampled for testing for phage presence at least once after 24 h of incubation. Sampling consisted of removing 180 µl of the culture, to which 20 µl of chloroform were added. After vortexing, the mixtures, 10 µl drops were pipetted on top of double-agar plates made using the hosts. For all the direct plating isolations, CaCl_2_ and MgSO_4_ were added to the cultures, at a final concentration of 1mM each. Addition of these chemicals were made in some of the enrichment cultures. Every plate was carefully checked for the appearance of plaques and considered negative once no plaque or lysis was detected after 24 h of incubation at 37 °C.

### Host range analysis

Host range analysis was performed by adding drops of phage-containing solutions to the top of double agar plates prepared with the appropriated host. Each host was tested once against each of the phages. For each phage four drops were tested, consisting of tenfold dilutions of the stocks. The plates were checked for the presence of phage plaques following an incubation of 24 h at 37 °C. Whenever possible, plaques were counted to determine a titre of the phage. If uncountable, the sample was considered positive if clear lysis appeared in the lowest dilution. If no lysis was observed even in the first dilution the result was recorded as negative.

### *S. borealis* HUS23 growth curve and biofilm Inhibition assay

Growth curves of *S. borealis* Hus23 in the presence of phage ISP were made by incubating the bacteria and phage together in 96-well plates at 37 °C with the OD_600_ of the cultures being measured every 30 min for a period of 24 h in a BioTek Synergy H1 Hybrid Reader (Agilent, Santa Clara, USA). The bacteria inoculum was 0.01 OD_600_ and the phage inoculum was either 1000 or 10 PFU per well. Experiments were performed in quadruplicates in either the presence of 1% glucose or in plain TSB media.

Biofilm formation was measured at the 24-hour time point by rinsing all wells with distilled water followed by drying the biomass at 55 °C for one hour and staining the biofilm with 0.1% crystal violet for five minutes. The excess crystal violet was removed by three washes made with distilled water. Then the crystal violet retained by the biofilm was dissolved in ethanol and measured in a plate reader at OD_600_.

### Phage ISP adaptation to *S. borealis* HUS23

Three sequential passages of the phage ISP in the *S. borealis* Hus23 host were made. The original phage received from Belgium was propagated once in the *S. aureus* PS47 host (passage 0) and used as starting point for the adaptation process. The first passage was made by serially diluting the passage 0 stock from 1- to −8 and adding five µl drops of each dilution to a double agar plate containing a confluent lawn of either *S. aureus* PS47 or *S. borealis* Hus23. This was done in triplicates. The plates were incubated overnight at 37 °C and plaques were counted to measure the titre in both hosts. For the second passage, one plaque was picked from each replicate in the *S. borealis* Hus23 plate, resuspended in 100 µl of TSB, and diluted to repeat the process. The same procedure was repeated once more for the third passage.

## Results

### Prophages are common in *S. borealis*

**Fig. 1:**
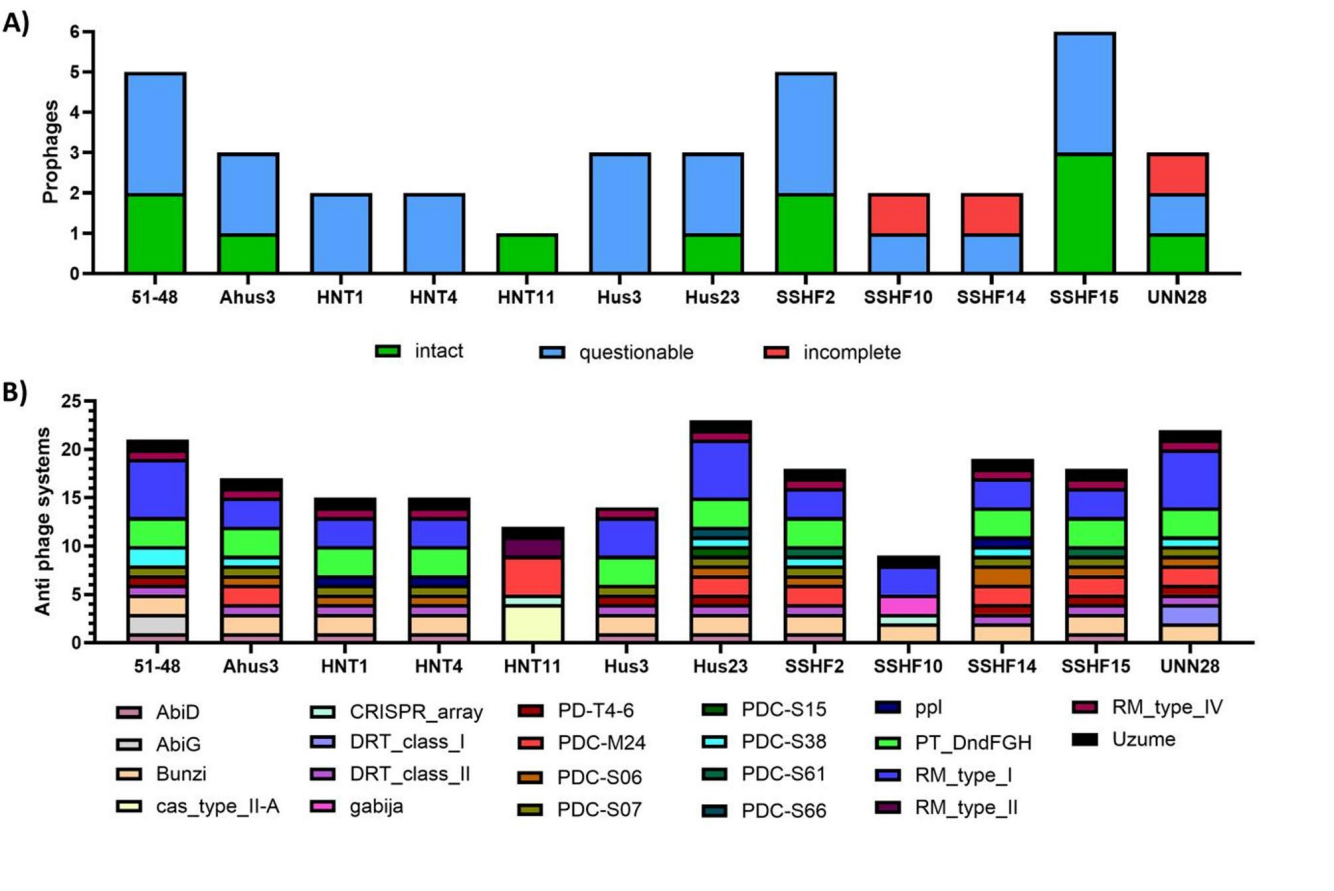
In silico detection of prophages and anti-phage systems in the genome of fifteen different *S. borealis* strains. Predicted prophage regions are shown in **A**. Predicted anti-phage defence systems are shown in **B**

To date, there is no information on prophage prevalence in *S. borealis* strains. We therefore aimed to identify prophages in the genomes of twelve different *S. borealis* strains in silico. All tested *S. borealis* strains contained evidence of prophages in their genomes (Fig. 1A). The strain with the most prophage hits was SSHF15, containing three intact and three questionable hits. The strain with least predicted prophages was HNT11, with only one intact hit. The *S. borealis* 51 − 48 type strain contains five putative prophage regions. Of these, three are considered questionable and two are considered complete with the maximum possible completeness score of 150. One of the predicted complete prophages in *S. borealis* 51 − 48 is closest to *S. epidermidis* phage CNPH82 [21]. The other is closest to *S. pseudintermedius* phage vB_SpsS_QT1 [22]. The complete overview of the PHASTEST results is shown in the Supplementary Table 1. We attempted to induce prophages in the *S. borealis* strain using three approaches: exposure to UV light, heat stress and nutrient depletion. No prophage was induced and thus these results are not shown.

### A large array of anti-phage defence systems is found in *S. borealis* genomes

We also described antiphage mechanisms in silico within *S. borealis* genomes. In total, twenty-two distinct anti-phage systems were predicted in the twelve *S. borealis* strains tested (Fig. 1B). *S. borealis* Hus23 was the strain with most unique anti-phage systems. *S. borealis* HNT11 and SSHF10 were the ones with least unique systems. The most prevalent systems were Bunzi, RM type I and Uzume. The AbiG, DRT class I, gabija, PDC-S15, PDC-S66 and RN type II systems were the least common, each appearing in only one strain each. The complete overview of the Padloc results is shown in the Supplementary Table 2.

### It was not possible to isolate phages capable of infecting *S. borealis* from Norwegian marine and wastewater samples

A large effort in isolating Norwegian staphylococci phages with a bias towards *S. borealis* was made during 2023. In total, six species of *Staphylococcus*, including twelve isolates of *S. borealis*, were used as hosts. The samples used for phage isolation were mostly wastewater samples collected from a sewage treatment facility in Tromsø (seventeen samples, coordinates 69.67527655551277, 18.978643977541036). Marine samples collected near the Tromsø harbour were also used (six samples, approximated coordinates 69.65800472705355, 18.98012132996835). All samples were filtered using 0.22 μm syringe filters and were kept cold from collection until use. In total, 258 combinations of strains and samples were made, of which 180 were tested for phage presence by direct plating and 78 went through the process of enrichment prior to plating. A compilation of the strains and samples used is shown in the Supplementary Table 3. Despite all attempts, no reproducible phage lysis was observed. Direct plating of three different wastewater samples in the *S. borealis* Hus23 host resulted in faint plaque-like growth inhibition in a first passage, but we were not able to further expand this potential virus or reproduce the finding with additional platings and thus these were also considered negative.

### Staphylococci phages from abroad infects Norwegian Staphylococci strains

In the absence of Norwegian phages to test, we decided to test the host range of three previously described, genetically diverse staphylococcal phages against a collection of *S. borealis* and other staphylococcal strains. The three phages used were *Kayvirus* ISP [16]*Rosenblumvirus* Huma [17] and *Silviavirus* Romulus [18]. Host range analysis was made using plaque assays with nine strains of *S. aureus*, twelve strains of *S. borealis*, one strain of *S. capitis*, four strains of *S. epidermidis*, twenty-four strains of *S. haemolyticus* and one strain of *S. lugdunensis*. The three phages were tested against all 51 host strains. The host range results are shown in Table 1. Phages Huma and Romulus had the same host range, infecting five of the nine *S. aureus* strains tested. Phage ISP was able to infect the same five *S. aureus* strains, but additionally also infected the *S. capitis* strain and the *S. borealis* Hus23 strain, making it the first recorded phage capable of infecting *S. borealis*. Whenever possible, the efficiency of plating (EOP) was calculated for phage ISP using the titre in *S. aureus* PS47 as reference. The highest EOP was in *S. capitis* (55.56%) and the lowest was on *S. borealis* Hus23 (5.56%). A compilation of the host range results is shown in Table 1.

### Phage ISP inhibits *S. borealis* Hus23 growth in vitro

**Fig. 2:**
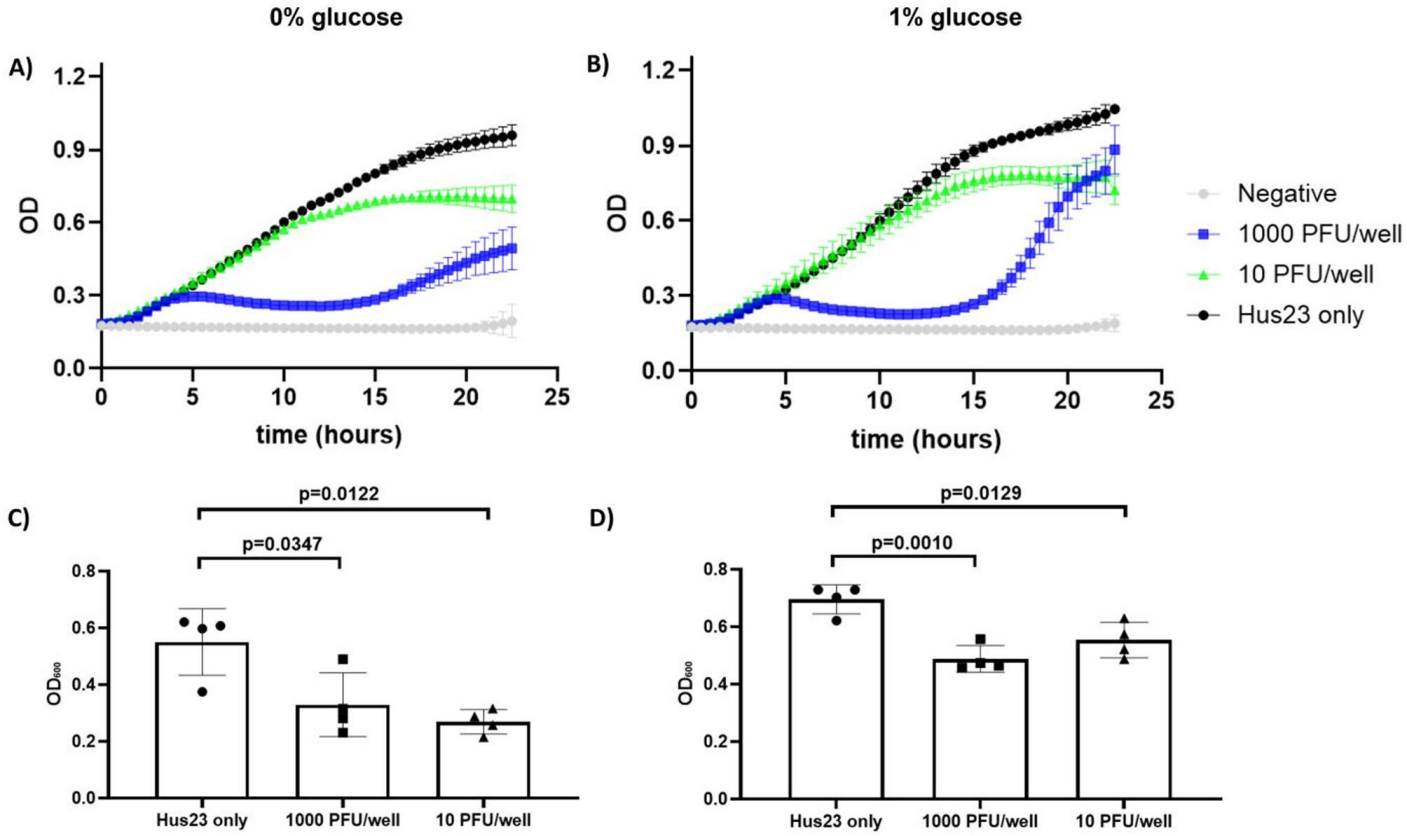
*S. borealis* Hus23 growth in the presence of phage ISP. Liquid cultures were monitored for 24 h after addition of both host and phage at the same time. OD readings of the cultures made in plain TSB media are shown in **A** and of the cultures made in TSB media supplemented with 1% glucose are shown in **B**. Biofilm formation of all cultures was measured at the endpoint (24 h) and the values are shown in **C** (plain TSB media) and in **D** (TSB media supplemented with 1% glucose). Each condition was tested in quadruplicates. The values in **A** and **B** are plotted as the average plus standard deviation of each collected data point. In **C** and **D** the individual replicates plus the standard deviation are shown in each bar

Following the finding that phage ISP is capable of infecting *S. borealis* in plates, we tested whether the phage can also inhibit growth of the host in liquid cultures. Growth curves of *S. borealis* Hus23 were made in the presence of two fixed amounts of phage ISP. Both conditions were tested in TSB media alone and in TSB media supplemented with 1% glucose as an enhancer of biofilm formation. The effect of phage ISP was clear in the two different concentrations used. The highest viral inoculum used (1000 PFU/well) limited bacterial growth until around 15 h of incubation, which was the time when the bacteria started to recover likely due to appearance of resistant cells. The effect of the lowest ISP inoculum (10 PFU/well) limited the maximum growth of the bacteria (Fig. 2A and B). All four phage-containing curves were significantly different from *S. borealis* only curves (Wilcoxon matched-pairs signed rank test, *p* < 0.0001 for 1000 PFU/well in 0% or 1% glucose, *p* < 0.0001 for 10 PFU/well in 0% glucose, *p* = 0.0002 for 10 PFU/well in 1% glucose). Similar patterns were noted in the cultures with or without glucose. However, the bacterial recovery was more pronounced in the highest concentration of phages in the presence of glucose (Fig. 2B). The amount of biofilm formed in the cultures was measured at the 24-hour time point. On average the highest concentration of ISP blocked 47% of the biofilm formation in the absence of glucose and 34% in the presence of glucose. The lowest concentration blocked 60% and 23% in the absence or presence of glucose, respectively. When compared to *S. borealis* only curves, all phage-containing curves significantly reduced biofilm formation (unpaired t-test with Welch correction, specific *p* values shown in Fig. 2C and D).

### Phage ISP can be adapted to better infect *S. borealis* Hus23

**Fig. 3:**
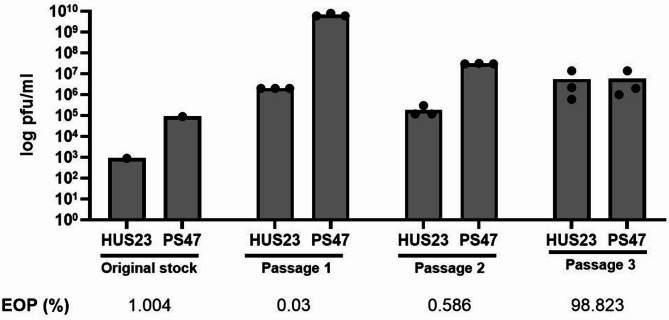
Adaptation of the phage ISP in *S. borealis* Hus23. The bars indicate the phage titre over the process of adaptation. The EOP is indicated below each passage and was calculated by considering the titre in the original *S. aureus* PS47 host as 100% in the corresponding passage. The three passages were made in triplicates

Sequential passages of phage ISP in the *S. borealis* Hus23 host were made to test whether the phage can be adapted to the new host. Three passages of the phage were made in the *S. borealis* Hus23 host and phage titres of each were measured in either the *S. borealis* Hus23 new host or in the original propagation strain *S. aureus* PS47. A clear improvement of the phage EOP after the three passages was seen (Fig. 3). When the original stock received from Belgium was titrated in both strains, the EOP in *S. borealis* Hus23 was only 1%, even lower than the EOP determined in the host range analysis. It was even lower after one and two passages in *S. borealis* Hus23 (0.03 and 0.586% respectively). However, after the third passage in *S. borealis* Hus23 the EOP was close to 99%, evidencing a successful adaptation to the new host. We attempted to infect the *S. borealis* 51 − 48 type strain with the ISP phage adapted in *S. borealis* Hus23 after the three passages, but no lysis was seen, indicating that the adaptation was specific to the Hus23 host.

## Discussion

Staphylococcal infections are on the rise and present a threat to humankind. A recent report from the World Health Organization concluded that *S. aureus* alone was the pathogen with the highest increase in deaths associated and attributable to AMR from 1990 to 2021 [23]. The description of the new species *S. borealis*, with some strains isolated from human patients, shows that new staphylococcal threats may appear and alternative or complementary approaches to antibiotic treatments are needed.

In this manuscript, we focused on understanding phage infections in *S. borealis*. No local Norwegian phages capable of infecting *S. borealis* were found in an isolation effort made surrounding the city of Tromsø. It is important to note that phages against other bacterial species unrelated to staphylococci were isolated from some of the same samples used in this study, evidencing that viable phages were present in the samples and reinforcing the finding that *S. borealis*-specific phages were harder to obtain. Phages against other coagulase-negative *Staphylococcus* are known, mostly isolated from European wastewater sources [15]. Out of 206 phages with deposited genomes infecting this group, 40% infects *S. epidermidis*. Many coagulase-negative species, including *S. borealis*, are still without phages associated to them.

We were able to record a phage infecting an *S. borealis* strain by testing the host range of three already described staphylococcal phages using a cohort of Norwegian staphylococcal strains. While *Rosenblumvirus* Huma and *Silviavirus* Romulus infected only *S. aureus* strains, *Kayvirus* ISP was able to also infect *S. capitis* and *S. borealis* Hus23. Phages Romulus and ISP were already compared to each other in another study focused on *S. aureus* causing mastitis in cattle. One of the two Norwegian strains tested in that study was infected by Romulus and the other by ISP [24]. Phage Huma was isolated in Iran [17]while phage Romulus was isolated in Belgium and is known to have a narrower host range when compared to phage ISP [18]. Phage ISP has an interesting history. Originally isolated on the 1920 s in Georgia, it became part of the Intravenous Staphylococcal Phage (ISP) preparation of the Eliava Institute, from which it was re-isolated in the 1970 s [16, 25]– [26]. Phage ISP is known to possess a broad host range [27] and is widely used in modern phage therapy clinical cases [13]. Phage ISP infection in *S. borealis* was explored further and we also showed that the phage limits *S. borealis* Hus23 growth in liquid cultures even at a low dose. An effect on the formation of biofilm was also recorded, but since phage ISP is known to attack planktonic cells only [28]we did not test its effect on mature biofilms.

It is curious that phage ISP infects only the *S. borealis* Hus23 strain and not the other thirteen *S. borealis* nor the twenty-four *S. haemolyticus* strains tested, despite the well documented ability of other kayviruses to lyse coagulase negative staphylococci [12, 29–35]. Interestingly, three of the samples tested during the phage isolation effort resulted in faint lysis on the *S. borealis* Hus23 strain, but these could not be reproduced further. *S. borealis* Hus23 has three predicted questionable prophages in its genome, two of them shared with the *S. borealis* 51 − 48 type strain. Of all the tested *S. borealis* strains, Hus23 was the one with the largest number of predicted anti-phage defence mechanisms, with the PDC-S15 and PDC-S66 systems being found only in this isolate. These ‘phage defence candidates’ are still uncharacterized, meaning that it is not possible to correlate their presence with an increased susceptibility to phage ISP. A correlation between the total abundance of phage defence systems and viral abundance has been determined by metagenomic analyses [36]suggesting that the *S. borealis* Hus23 might have evolved under stronger viral pressure than other *S. borealis* lineages. Although a positive correlation between the number of phage defence systems and phage protection has been shown in some models [37]this correlation has a marginal role in others [38]which could be the case for *S. borealis* Hus23. Numerous genetic determinants not linked to phage defence systems but important for phage host-range are known for *S. aureus* strains [39]adding to the seemingly complex combination of host, phage and epigenetic patters that determines the host range of *Staphylococcus* phages [40]. In a study focused on susceptibility of hosts to phage ISP, it was concluded that host phylogeny accounts for most ISP susceptibility [41]. However, this is not true in our case, since only one of twelve *S. borealis* strains was infected by this phage.

The EOP of phage ISP in *S. borealis* Hus23 was the lowest during the host range analysis and when the phage stock was plated and the titre compared between the *S. aureus* PS47 propagation host and the *S. borealis* Hus23 strain. In a simple evolution experiment we were able to increase the EOP of phage ISP from low to almost 100% by three sequential passages in *S. borealis* Hus23. This shows that adaptation of the phage to better infect *S. borealis* is possible using in vitro conditions, similar to what has been shown to another Kayvirus^42^. Adaptation of phage ISP to a *S. epidermidis* strain, leading to gain in therapeutic value but loses in host range, was already shown in a clinical case [13]. Adaptation of staphylococci phages to clinical strains isolated from patients prior to phage therapy was also recommended for successful outcomes in the early 20th century [10].

## Conclusions

To the best of our knowledge, we here describe the first evidence of phage ISP infection in *S. capitis* and in *S. borealis*, expanding the host range of this valuable phage used in phage therapy. Phage ISP was also shown to be efficient in reducing *S. borealis* Hus23 growth in liquid and was adapted to this new host with only three passages in vitro. This shows that while no specific *S. borealis* phage is found in this search, already known staphylococci phages have potential to be evaluated and adapted for clinical cases. Phage ISP serves as a link between classical and modern phage use, being isolated in the beginning of the phage therapy golden years and now becoming the first phage recorded that can kill the recently described *S. borealis* species.

## Supplementary Information

Supplementary Material 1.

Supplementary Material 2.

Supplementary Material 3.

## Data Availability

The datasets used and/or analysed during the current study are available from the corresponding author on reasonable request.
